# Supplementary material for: Portable digital devices for paediatric height and length measurement: A scoping review and target product profile matching analysis
Source: PLoS One. 2023 Jul 26;18(7):e0288995. doi: 10.1371/journal.pone.0288995 (PMC10370750; doi:10.1371/journal.pone.0288995)
Supplement: S1 Appendix — (DOCX) [file pone.0288995.s002.docx]

Appendix 1: Search Strategies

Medline search:

| 1 | body mass index/ or body size/ or body height/ or waist-height ratio/ |
| --- | --- |
| 2 | Body Height/ |
| 3 | Anthropometry/ |
| 4 | 1 or 2 or 3 |
| 5 | adolescent/ or child/ or child, preschool/ or infant/ or infant, newborn/ |
| 6 | Pediatrics/ or neonatology/ |
| 7 | adolescent development/ or child development/ |
| 8 | 5 or 6 or 7 |
| 9 | Lasers/ |
| 10 | Mobile Applications/ |
| 11 | Imaging, Three-Dimensional/ |
| 12 | Software/ |
| 13 | Smartphone/ |
| 14 | 9 or 10 or 11 or 12 or 13 |
| 15 | 4 and 8 and 14 |
| 16 | limit 15 to last 30 years |
| 17 | ((portable or handheld or hand-held or laser*) adj4 (anthropomet* or anthropomorph* or morphomet)).mp. |
| 18 | ((3D or 3Dimension* or "3 D" or "3 dimension*") adj4 (body or height or length) adj3 measure*).mp. |
| 19 | ((3D or 3Dimension* or "3 D" or "3 dimension*") adj4 (body measure* or height measure* or length measure*)).mp. |
| 20 | ((digital or electronic or mobile or smartphone) adj4 (anthropomet* or anthropomorph* or morphomet)).mp. |
| 21 | ((digital or electronic or mobile or smartphone) adj4 (body measure* or height measure* or length measure*)).mp. |
| 22 | ((portable or handheld or hand-held or laser*) adj4 (body measure* or height measure* or length measure*)).mp. |
| 23 | 17 or 18 or 19 or 20 or 21 or 22 |
| 24 | (child* or adolesc* or infan* or p?ediatric* or toddler* or newborn* or neonat* or baby or babies or schoolchild* or preschool* or pre-school* or teen*).mp. |
| 25 | 23 and 24 |
| 26 | limit 25 to yr="1992 -Current" |
| 27 | 16 or 26 |

Embase search:

| 1 | anthropometric parameters/ or anthropometry/ |
| --- | --- |
| 2 | body composition/ |
| 3 | body height/ |
| 4 | body build/ |
| 5 | 1 or 2 or 3 or 4 |
| 6 | child/ |
| 7 | pediatrics/ |
| 8 | infant/ |
| 9 | adolescent/ |
| 10 | adolescent health/ |
| 11 | infant nutrition/ |
| 12 | school child/ |
| 13 | child health/ |
| 14 | preschool child/ |
| 15 | child nutrition/ |
| 16 | hospitalized infant/ |
| 17 | 6 or 7 or 8 or 9 or 10 or 11 or 12 or 13 or 14 or 15 or 16 |
| 18 | devices/ |
| 19 | laser/ |
| 20 | morphometry/ |
| 21 | three-dimensional imaging/ |
| 22 | portable equipment/ or portable computed tomography scanner/ or portable ultrasound scanner/ |
| 23 | medical device/ |
| 24 | anthropometric device/ |
| 25 | smartphone/ or mobile phone/ |
| 26 | 18 or 19 or 20 or 21 or 22 or 23 or 24 or 25 |
| 27 | 5 and 17 and 26 |
| 28 | limit 27 to last 30 years |
| 29 | ((portable or handheld or hand-held or laser*) adj4 (anthropomet* or anthropomorph* or morphomet)).mp. |
| 30 | ((3D or 3Dimension* or "3 D" or "3 dimension*") adj4 (body or height or length) adj3 measure*).mp. |
| 31 | ((3D or 3Dimension* or "3 D" or "3 dimension*") adj4 (body measure* or height measure* or length measure*)).mp. |
| 32 | ((digital or electronic or mobile or smartphone) adj4 (anthropomet* or anthropomorph* or morphomet)).mp. |
| 33 | ((digital or electronic or mobile or smartphone) adj4 (body measure* or height measure* or length measure*)).mp. |
| 34 | ((portable or handheld or hand-held or laser*) adj4 (body measure* or height measure* or length measure*)).mp. |
| 35 | 29 or 30 or 31 or 32 or 33 or 34 |
| 36 | (child* or adolesc* or infan* or p?ediatric* or toddler* or newborn* or neonat* or baby or babies or schoolchild* or preschool* or pre-school* or teen*).mp. |
| 37 | 35 and 36 |
| 38 | limit 37 to yr="1992 -Current" |
| 39 | 28 or 38 |

Global Health Search:

(("height" OR "body mass index" OR "body measurements" OR "height-weight ratio" OR "anthropometry" OR "anthropometric dimensions" OR "anthropometric measurements") AND ("child health" OR "child development" OR "child nutrition" OR "children" OR "infant children" OR "infant development" OR "infant nutrition" OR "infants" OR "adolescent" OR "adolescent development" OR "adolescents" OR "teenager" OR "teenagers") AND ("ultrasonic devices" OR "equipment" OR "lasers" OR "laser beams" OR "computer software" OR "mobile applications" OR "computers" OR "instrumentation" OR "mechanization" OR "automation") AND yr:[1991 TO 2023])

CINAHL Search:
S13 ((S5 OR S6 OR S7 OR S8 OR S9 OR S10) AND (S3 OR S4)) AND (S1 OR S2) Limiters - Published Date: 19910101-20211231

S12 (S5 OR S6 OR S7 OR S8 OR S9 OR S10) AND (S3 OR S4)

S11 S5 OR S6 OR S7 OR S8 OR S9 OR S10

S10 (MH "Imaging, Three-Dimensional+") OR (MH "Photography+")

S9 (MH "Biosensors")
S8 (MH "Equipment Reliability") OR (MH "Portable Equipment")

S7 (MH "Mobile Applications")

S6 (MH "Lasers+")
S5 (MH "Imaging, Three-Dimensional+") OR (MH "Ultrasonography+")
S4 (MH "Nutritional Assessment")

S3 (MH "Body Height") OR (MH "Anthropometry+") OR (MH "Body Size") OR (MH "Body Mass Index")
S2 (MH "Hospitals, Pediatric") OR (MH "Pediatric Care+") OR (MH "Pediatrics+")

S1 (MH "Infant+") OR (MH "Child+") OR (MH "Infant, Newborn") OR (MH "Adolescence+")

Appendix 2: Table scoring devices against the UNICEF target product profile

|  | **SCANTIFY [1,24]** | **Leica Disto D2 [7, 20, 39]** | **AutoAnthro *use in controlled environment [16, 21]** | **AutoAnthro *use in field [25, 26]** | **Gulliver [33]** | **GLM40 [28, 40]** | **Cannon Digital Camera [22]** | **Digital Camera [18]** | **P2B2 [29, 30]** | **Optisizer [27]** | **Coolpix [23]** | **GLM30 [21]** | **PePA* [34]** |
| --- | --- | --- | --- | --- | --- | --- | --- | --- | --- | --- | --- | --- | --- |
| **Operational Functional Requirements** |  |  |  |  |  |  |  |  |  |  |  |  |  |
| 1. Measures height/length |  |  |  |  |  |  |  |  |  |  |  |  |  |
| 1. Used for growth monitoring |  |  |  |  |  |  |  |  |  |  |  |  |  |
| 1. Stored in wide ranging climates |  |  |  |  |  |  |  |  |  |  |  |  |  |
| 1. Used recumbent or standing |  |  |  |  |  |  |  |  |  |  |  |  |  |
| 1. Power requirements |  |  |  |  |  |  |  |  |  |  |  |  |  |
| 1. Auto shut off battery |  |  |  |  |  |  |  |  |  |  |  |  |  |
| 1. Notifications |  |  |  |  |  |  |  |  |  |  |  |  |  |
| 1. Calibration |  |  |  |  |  |  |  |  |  |  |  |  |  |
| 1. Output |  |  |  |  |  |  |  |  |  |  |  |  |  |
| 1. Data storage |  |  |  |  |  |  |  |  |  |  |  |  |  |
| 1. Time for set up |  |  |  |  |  |  |  |  |  |  |  |  |  |
| **Performance Requirements** |  |  |  |  |  |  |  |  |  |  |  |  |  |
| 1. Accuracy of measurements |  |  |  |  |  |  |  |  |  |  |  |  |  |
| 1. Accuracy of recordings |  |  |  |  |  |  |  |  |  |  |  |  |  |
| 1. Precision |  |  |  |  |  |  |  |  |  |  |  |  |  |
| 1. Range |  |  |  |  |  |  |  |  |  |  |  |  |  |
| 1. Timing of result |  |  |  |  |  |  |  |  |  |  |  |  |  |
| **Product Requirements** |  |  |  |  |  |  |  |  |  |  |  |  |  |
| 1. Storage life |  |  |  |  |  |  |  |  |  |  |  |  |  |
| 1. Operational life |  |  |  |  |  |  |  |  |  |  |  |  |  |
| 1. Operational climactic conditions |  |  |  |  |  |  |  |  |  |  |  |  |  |
| 1. Durability |  |  |  |  |  |  |  |  |  |  |  |  |  |
| 1. Physical characteristics |  |  |  |  |  |  |  |  |  |  |  |  |  |
| 1. Maintenance |  |  |  |  |  |  |  |  |  |  |  |  |  |
| **User Requirements** |  |  |  |  |  |  |  |  |  |  |  |  |  |
| 1. Portability |  |  |  |  |  |  |  |  |  |  |  |  |  |
| 1. Assembly/set up |  |  |  |  |  |  |  |  |  |  |  |  |  |
| 1. Operational use |  |  |  |  |  |  |  |  |  |  |  |  |  |
| 1. Output display |  |  |  |  |  |  |  |  |  |  |  |  |  |
| 1. Data download |  |  |  |  |  |  |  |  |  |  |  |  |  |
| 1. Training requirements |  |  |  |  |  |  |  |  |  |  |  |  |  |
| **Supply Chain Requirements** |  |  |  |  |  |  |  |  |  |  |  |  |  |
| 1. Packaging |  |  |  |  |  |  |  |  |  |  |  |  |  |
| 1. Environmental footprint |  |  |  |  |  |  |  |  |  |  |  |  |  |
| **Commercialization Requirements** |  |  |  |  |  |  |  |  |  |  |  |  |  |
| 1. Regulatory approvals |  |  |  |  |  |  |  |  |  |  |  |  |  |
| 1. Safety requirements |  |  |  |  |  |  |  |  |  |  |  |  |  |
| 1. Target price |  |  |  |  |  |  |  |  |  |  |  |  |  |
| 1. Children’s rights principles |  |  |  |  |  |  |  |  |  |  |  |  |  |

Key:

| UNICEF criteria met to ideal standard |  |
| --- | --- |
| UNICEF criteria met to minimum standard |  |
| UNICEF criteria NOT met |  |
| CRITERIA not addressed in the literature |  |

* Pediatric Platform for Anthropometry

Appendix 3: Comparison of prospective validation studies

| \|  \| Author \| Kessing et al [24] \| Andrews et al [1] \| Bauman et al [20] Bauman et al [27] \| Bougma et all [25] \| Conkle et al [21] \| Glock et al [33] \| Leidman et al [26] \| Mayol-Kreiser, et al [28] \| Penders et al [22] \| Sokolover et al [18] \| Syafiq et al [29, 30] \| Watt et al [19] \| Wetzel et al [27] \| \| --- \| --- \| --- \| --- \| --- \| --- \| --- \| --- \| --- \| --- \| --- \| --- \| --- \| --- \| --- \| \| Title and abstract \| Identify in title or abstract that interrater/intrarater reliability or agreement was investigated \| Y \| Y \| Y \| Y \| Y \| N \| Y \| Y \| Y \| Y \| Y \| N \| Y \| \| Introduction \| Name and describe the diagnostic or measurement device of interest explicitly. \| Y \| Y \| Y \| Y \| Y \| Y \| Y \| Y \| Y \| N \| Y \| Y \| Y \| \| Specify the subject or population of interest \| Y \| Y \| Y \| Y \| Y \| Y \| Y \| Y \| Y \| Y \| Y \| Y \| Y \| \| Specify the rater population of interest (if applicable) \| Y \| Y \| Y \| Y \| Y \| N \| Y \| Y \| Y \| Y \| Y \| Y \| Y \| \| Describe what is already known about the reliability and agreement and provide a rational for the study (if applicable) \| - \| - \| Y \| Y \| Y \| N \| Y \| Y \| Y \| Y \| Y \| N \| Y \| \| Methods \| Explain how the sample size was chosen. State the determined number of raters, subjects/objects and replicate observations. \| N \| N \| N \| Y \| Y \| N \| Y \| N \| N \| N \| N \| N \| N \| \| Describe the sampling method \| N \| N \| Y \| Y \| Y \| N \| Y \| N \| Y \| Y \| N \| Y \| N \| \| Describe the measurement rating processes (i.e., time interval between repeated measurements, availability of clinical information, blinding) \| Y \| Y \| Y \| Y \| Y \| N \| Y \| Y \| Y \| Y \| Y \| Y \| Y \| \| State whether measurements/ratings were conducted independently \| Y \| Y \| Y \| Y \| Y \| N \| Y \| Y \| Y \| Y \| Y \| Y \| Y \| \| Describe statistical analysis \| Y \| Y \| Y \| Y \| Y \| N \| Y \| Y \| Y \| Y \| Y \| Y \| Y \| \| Results \| State the actual number of raters and subjects/objects which were included and the number of replicate observations conducted \| Y \| Y \| Y \| Y \| Y \| Y \| Y \| Y \| Y \| Y \| Y \| Y \| Y \| \| Describe the sample characteristics of raters and subjects (i.e., training and experience) \| N \| N \| Y \| Y \| Y \| N \| Y \| N \| Y \| Y \| N \| N \| Y \| \| Report estimates of reliability and agreement including measures of statistical uncertainty \| Y \| Y \| Y \| Y \| Y \| N \| Y \| Y \| Y \| Y \| Y \| Y \| Y \| \| Discussion \| Discuss the practical relevance of results \| Y \| Y \| Y \| Y \| Y \| Y \| Y \| Y \| Y \| Y \| Y \| Y \| Y \| \| Axillary material \| Provide detailed results if possible \| Y \| Y \| Y \| Y \| Y \| N \| Y \| Y \| Y \| Y \| Y \| N \| Y \| \| Total Y/15 \|  \| 11 \| 11 \| 14 \| 15 \| 15 \| 4 \| 15 \| 12 \| 14 \| 13 \| 13 \| 10 \| 13 \| |
| --- | --- | --- | --- | --- | --- | --- | --- | --- | --- | --- | --- | --- | --- | --- | --- | --- | --- | --- | --- | --- | --- | --- | --- | --- | --- | --- | --- | --- | --- | --- | --- | --- | --- | --- | --- | --- | --- | --- | --- | --- | --- | --- | --- | --- | --- | --- | --- | --- | --- | --- | --- | --- | --- | --- | --- | --- | --- | --- | --- | --- | --- | --- | --- | --- | --- | --- | --- | --- | --- | --- | --- | --- | --- | --- | --- | --- | --- | --- | --- | --- | --- | --- | --- | --- | --- | --- | --- | --- | --- | --- | --- | --- | --- | --- | --- | --- | --- | --- | --- | --- | --- | --- | --- | --- | --- | --- | --- | --- | --- | --- | --- | --- | --- | --- | --- | --- | --- | --- | --- | --- | --- | --- | --- | --- | --- | --- | --- | --- | --- | --- | --- | --- | --- | --- | --- | --- | --- | --- | --- | --- | --- | --- | --- | --- | --- | --- | --- | --- | --- | --- | --- | --- | --- | --- | --- | --- | --- | --- | --- | --- | --- | --- | --- | --- | --- | --- | --- | --- | --- | --- | --- | --- | --- | --- | --- | --- | --- | --- | --- | --- | --- | --- | --- | --- | --- | --- | --- | --- | --- | --- | --- | --- | --- | --- | --- | --- | --- | --- | --- | --- | --- | --- | --- | --- | --- | --- | --- | --- | --- | --- | --- | --- | --- | --- | --- | --- | --- | --- | --- | --- | --- | --- | --- | --- | --- | --- | --- | --- | --- | --- | --- | --- | --- | --- | --- | --- | --- | --- | --- | --- | --- | --- | --- | --- | --- | --- |

*Y=YES, N=NO

Appendix 4: MMAT scores

| Appendix table MMAT scores   \| Author \|  \| Andrews et al [1] \| Kessing et al [24] \| Bauman et al [20] Bauman et al [27] \| Bougma et all [25] \| Conkle et al [21] \| Glock et al  [33] \| Leidman et al [26] \| Mayol-Kreiser, et al [28] \| Penders et al [22] \| Sokolover et al [18] \| Syafiq et al [29, 30] \| Watt et al [19] \| Wetzel et al [27] \| Jefferds et al [17] \| Conkle et al [16] \| \| --- \| --- \| --- \| --- \| --- \| --- \| --- \| --- \| --- \| --- \| --- \| --- \| --- \| --- \| --- \| --- \| --- \| \| Screening 1:  Are there clear research questions? \|  \| Y \| Y \| Y \| Y \| Y \| Y \| Y \| Y \| Y \| Y \| Y \| Y \| Y \|  \|  \| \| Screening 2:  Do the collected data allow to address the research questions? \|  \| Y \| Y \| Y \| Y \| Y \| Y \| Y \| Y \| Y \| Y \| Y \| Y \| Y \|  \|  \|  \| \| Quantitative nonrandomized \|  \|  \|  \|  \|  \|  \|  \|  \|  \|  \|  \|  \|  \|  \|  \|  \| \| Are the participants representative of the target population? \|  \| Y \| Y \| Y \| Y \| Y \| Y \| Y \| Y \| Y \| Y \| Y \| Y \| Y \|  \|  \| \| Are measurements appropriate regarding both the outcome and intervention (or exposure)? \|  \| Y \| Y \| Y \| Y \| Y \| N \| Y \| Y \| Y \| Y \| Y \| N \| Y \|  \|  \| \| Are there complete outcome data? \|  \| Y \| Y \| Y \| Y \| Y \| N \| Y \| Y \| Y \| Y \| Y \| N \| Y \|  \|  \| \| Are the confounders accounted for in the design and analysis? \|  \| N \| N \| Y \| Y \| Y \| N \| Y \| Y \| Y \| Y \| Y \| N \| N \|  \|  \| \| During the study period, is the intervention administered (or exposure occurred) as intended? \|  \| Y \| Y \| Y \| Y \| Y \| Y \| Y \| Y \| Y \| Y \| Y \| Y \| Y \|  \|  \| \| Mixed methods \|  \|  \|  \|  \|  \|  \|  \|  \|  \|  \|  \|  \|  \|  \|  \|  \| \| Is there an adequate rationale for using a mixed methods design to address the research question? \|  \|  \|  \|  \|  \|  \|  \|  \|  \|  \|  \|  \|  \|  \| Y \| Y \| \| Are the different components of the study effectively integrated to answer the research question? \|  \|  \|  \|  \|  \|  \|  \|  \|  \|  \|  \|  \|  \|  \| Y \| Y \| \| Are the outputs of the integration of qualitative and quantitative components adequately interpreted? \|  \|  \|  \|  \|  \|  \|  \|  \|  \|  \|  \|  \|  \|  \| Y \| Y \| \| Are divergences and inconsistencies between quantitative and qualitative results adequately addressed? \|  \|  \|  \|  \|  \|  \|  \|  \|  \|  \|  \|  \|  \|  \| Y \| Y \| \| Do the different components of the study adhere to the quality criteria of each tradition of the methods involved? \|  \|  \|  \|  \|  \|  \|  \|  \|  \|  \|  \|  \|  \|  \| Y \| Y \| \| Overall score \|  \| 80% \| 80% \| 100% \| 100% \| 100% \| 40% \| 100% \| 100% \| 100% \| 100% \| 100% \| 40% \| 80% \| 100% \| 100% \|   *Y=YES, N=NO |
| --- | --- | --- | --- | --- | --- | --- | --- | --- | --- | --- | --- | --- | --- | --- | --- | --- | --- | --- | --- | --- | --- | --- | --- | --- | --- | --- | --- | --- | --- | --- | --- | --- | --- | --- | --- | --- | --- | --- | --- | --- | --- | --- | --- | --- | --- | --- | --- | --- | --- | --- | --- | --- | --- | --- | --- | --- | --- | --- | --- | --- | --- | --- | --- | --- | --- | --- | --- | --- | --- | --- | --- | --- | --- | --- | --- | --- | --- | --- | --- | --- | --- | --- | --- | --- | --- | --- | --- | --- | --- | --- | --- | --- | --- | --- | --- | --- | --- | --- | --- | --- | --- | --- | --- | --- | --- | --- | --- | --- | --- | --- | --- | --- | --- | --- | --- | --- | --- | --- | --- | --- | --- | --- | --- | --- | --- | --- | --- | --- | --- | --- | --- | --- | --- | --- | --- | --- | --- | --- | --- | --- | --- | --- | --- | --- | --- | --- | --- | --- | --- | --- | --- | --- | --- | --- | --- | --- | --- | --- | --- | --- | --- | --- | --- | --- | --- | --- | --- | --- | --- | --- | --- | --- | --- | --- | --- | --- | --- | --- | --- | --- | --- | --- | --- | --- | --- | --- | --- | --- | --- | --- | --- | --- | --- | --- | --- | --- | --- | --- | --- | --- | --- | --- | --- | --- | --- | --- | --- | --- | --- | --- | --- | --- | --- | --- | --- | --- | --- | --- | --- | --- | --- | --- | --- | --- | --- | --- | --- | --- | --- | --- | --- | --- | --- | --- | --- | --- | --- | --- | --- | --- | --- | --- | --- | --- | --- | --- | --- | --- | --- | --- | --- | --- | --- | --- | --- | --- | --- | --- | --- | --- | --- | --- | --- | --- | --- | --- | --- | --- | --- | --- | --- | --- | --- |

Appendix 5: List of full text articles excluded with reason for exclusion

| Title | Lead author | Journal | Year of publication | Reason for exclusion |
| --- | --- | --- | --- | --- |
| Accurate determination of height using an inexpensive measuring device | Diamond | Clinical paediatrics | 1994 | Studies using ineligible interventions, such as non-portable technology |
| An anthropometric survey using digital photogrammetry: a case study in Recife, Pernambuco, Brazil | Barros et | IOS press | 2012 | Adult population |
| Body shape and size in 6-year old children: Assessment by three-dimensional photonic scanning | Santos | International journal of obesity | 2016 | Studies measuring anthropometric outcomes other than height or length |
| Digital anthropometric evaluation of young children | Kennedy | Obesity | 2020 | Studies measuring anthropometric outcomes other than height or length |
| Determination of the precision and accuracy of morphological measurements using the KinectTM sensor: comparison with standard stereophotogrammetry | Bonnechere | Ergonomics | 2014 | Adult population |
| A comparison of manual anthropometric measurements with Kinect-based scanned measurements in terms of precision and reliability | Braganca | Work | 2018 | Adult population |
| Automated anthropometric phenotyping with novel Kinect-based three-dimensional imaging method: Comparison with a reference laser imaging system | Soileau | European Journal of Clinical Nutrition | 2016 | Studies using ineligible interventions, such as non-portable technology |
| Longitudinal anthropometry of children and adolescents using 3D-body scanning | Binder | PLoS ONE | 2018 | Studies using ineligible interventions, such as non-portable technology |
| Three-dimensional body scanning: a new method to estimate body surface area in neonates | Schloesser | Neonatology | 2011 | Studies measuring anthropometric outcomes other than height or length |
| Digital three-dimensional photogrammetry: Evaluation of anthropometric precision and accuracy using a Genex 3D camera system | Weinberg | Cleft Palate-Craniofacial Journal | 2004 | Studies measuring anthropometric outcomes other than height or length |
| Child t-shirt size data set from 3D body scanner anthropometric measurements and a questionnaire | Pierola | Data in brief | 2017 | Other |
| Optical imaging technology for body size and shape analysis: evaluation of a system designed for personal use | Kennedy | European journal of clinical nutrition | 2020 | Studies using ineligible interventions, such as non-portable technology |
| Validity assessment of a portable anthropometer to measure length in 24-month children from the 2015 Pelotas Birth Cohort | Flores | Public health nutrition | 2020 | Studies measuring anthropometric outcomes other than height or length |
| BMI compared with 3-dimensional body shape: The UK National Sizing Survey | Cole | American Journal of Clinical Nutrition | 2007 | Adult population |
| Child body shape measurement using depth cameras and a statistical body shape model | Park | Ergonomics | 2015 | Studies measuring anthropometric outcomes other than height or length |
| Anthropometry: Continued refinements and new developments of an ancient method | Heymsfield | American Journal of Clinical Nutrition | 2017 | Other |
| Roll up the tape? Laser and optical technologies improve paediatric weight estimation | Czarnecki | Resuscitation | 2020 | Studies measuring anthropometric outcomes other than height or length |
| ABase - A tool for the rapid assessment of anthropometric measurements on handheld computers | Molinari | American Journal of Medical Genetics | 2003 | Studies measuring anthropometric outcomes other than height or length |
| Whole-body three-dimensional photonic scanning: A new technique for obesity research and clinical practice | Ruto | International Journal of Obesity | 2008 | Studies measuring anthropometric outcomes other than height or length |
| Comparison of height measuring instruments for children used in two municipalities of Laguna, Philippines | Gordoncillo | Southeast Asian Journal of Tropical Medicine and Public Health | 2020 | Studies using ineligible interventions, such as non-portable technology |
| Parametric body shape model of standing children aged 3-11 years | Park | Ergonomics | 2015 | Other |
| Validation of a 3-dimensional photonic scanner for the measurement of body volumes, dimensions, and percentage body fat | Wang | American Journal of Clinical Nutrition | 2006 | Studies measuring anthropometric outcomes other than height or length |
| Technical note: Criterion validity of whole body surface area equations: a comparison using 3D laser scanning | Daniell | American journal of physical anthropology | 2012 | Adult population |
| Reliability of transportable instruments for assessment of infant length | Byrne | Journal of Nursing Measurement | 2002 | Studies using ineligible interventions, such as non-portable technology |
| Stunting: Challenges in estimating prevalence and potential solutions | Martorell | Annals of Nutrition and Metabolism | 2017 | Studies using ineligible interventions, such as non-portable technology |
| Improving the quality of child anthropometry: Manual anthropometry in the body imaging for nutritional assessment study (BINA) | Conkle | PLoS ONE | 2017 | Studies using ineligible interventions, such as non-portable technology |
| Children and adolescents' anthropometrics body composition from 3-D optical surface scans | Wong | Obesity | 2019 | Studies measuring anthropometric outcomes other than height or length |
| Body typing of children and adolescents using 3D-body scanning | Loeffler-Wirth | PLoS ONE | 2017 | Studies measuring anthropometric outcomes other than height or length |
| Validity of a 3-dimensional body scanner: comparison against a 4-compartment model and dual energy X-ray absorptiometry | Cabre | Applied Physiology, Nutrition & Metabolism | 2021 | Studies measuring anthropometric outcomes other than height or length |
| Validation study of a Kinect based body imaging system | Braganca | Work (Reading, Mass.) | 2017 | Adult population |
| Acceptability, precision and accuracy of 3D photonic scanning for measurement of body shape in a multi-ethnic sample of children aged 5-11 years? The SLIC study | Wells | PLoS ONE | 2015 | Studies measuring anthropometric outcomes other than height or length |
| SHAPECODER: a new method for visual quantification of body mass index in young children | Park | Pediatric Obesity | 2018 | Studies using ineligible interventions, such as non-portable technology |
| Nutritional Status of Children (5-18 Years) by Using Anthropometric Indices: A Cross-Sectional Study Among the Sartang and Miji, Lesser Known Tribes of Arunachal Pradesh, India | Asghar | Journal of Nepal Paediatric Society | 2022 | Studies using ineligible interventions, such as non-portable technology |
| The inter-tester reliability of anthropometric measurement with portable tools | McKenna | European Journal of Physiotherapy | 2013 | Studies using ineligible interventions, such as non-portable technology |
| Digital anthropometric evaluation of young children: comparison to results acquired with conventional anthropometry | Kennedy | European journal of clinical nutrition | 2021 | Studies measuring anthropometric outcomes other than height or length |
| Methodological aspects of the anthropometric assessment in the Brazilian National Survey on Child Nutrition (ENANI-2019): a population-based household survey | Anjos | Cadernos de Saúde Pública | 2021 | Studies using ineligible interventions, such as non-portable technology |
| Anthropometric Kit Development for Stunted Early Detection among Children Under-two Years Old: Providing a Portable Body Length Measurer | Wigati | Open Access Macedonian Journal of Medical Sciences | 2022 | Studies using ineligible interventions, such as non-portable technology |
| Body size measuring techniques enabling stress-free growth monitoring of extreme preterm infants inside incubators: A systematic review | van Gils | PLoS ONE | 2022 | Other |
