# Supplementary material for: Portable digital devices for paediatric height and length measurement: A scoping review and target product profile matching analysis
Source: PLoS One. 2023 Jul 26;18(7):e0288995. doi: 10.1371/journal.pone.0288995 (PMC10370750; doi:10.1371/journal.pone.0288995)
Supplement: Attachment — Submitted filename: response to reviewers.docx [file pone.0288995.s003.docx]

**Dear Alex Schaefer,**

**Many thanks for your response. We appreciate the time and thought taken to review our manuscript. The reviewers have provided valuable feedback which we feel has strengthened our manuscript, creating an informative review on portable height/length measurement devices. This topic has been highlighted by UNICEF as requiring innovation in order to improve nutritional surveillance and clinical assessment of children worldwide. We feel that this study adds depth to the current literature, enhancing the knowledge base and creating a platform for future innovation.**

**Kind Regards,**

**Tasmyn, on behalf of the study team**

1. Please ensure that your manuscript meets PLOS ONE's style requirements, including those for file naming. The PLOS ONE style templates can be found at 
<https://journals.plos.org/plosone/s/file?id=wjVg/PLOSOne_formatting_sample_main_body.pdf> and 
<https://journals.plos.org/plosone/s/file?id=ba62/PLOSOne_formatting_sample_title_authors_affiliations.pdf>

**Response: We have amended the manuscript to meet attached style guidelines**

2. Please complete a PRISMA-ScR checklist (available at <https://www.equator-network.org/wp-content/uploads/2018/09/PRISMA-ScR-Fillable-Checklist-1.docx>) and upload it as supplementary file.

**Response: We have attached the completed checklist as a supplementary file**

3. Thank you for stating the following financial disclosure: 
  "This research received no specific grant from any funding agency in the public, commercial or not-for-profit sectors. JPV is supported by a NHMRC Investigator Grant. All authors declare: no support from any organisation for the submitted work; no financial relationships with any organisations that might have an interest in the submitted work; no other relationships or activities that could appear to have influenced the submitted work."

At this time, please address the following queries:

a) Please clarify the sources of funding (financial or material support) for your study. List the grants or organizations that supported your study, including funding received from your institution. 
b) State what role the funders took in the study. If the funders had no role in your study, please state: “The funders had no role in study design, data collection and analysis, decision to publish, or preparation of the manuscript.”
c) If any authors received a salary from any of your funders, please state which authors and which funders.
d) If you did not receive any funding for this study, please state: “The authors received no specific funding for this work.”

Please include your amended statements within your cover letter; we will change the online submission form on your behalf.

**Response: Amended statement as follows**

***The authors received no specific funding for this work.***

4. Please ensure that you include a title page within your main document. You should list all authors and all affiliations as per our author instructions and clearly indicate the corresponding author.

**Response: We have amended the manuscript accordingly**

5. Please include captions for your Supporting Information files at the end of your manuscript, and update any in-text citations to match accordingly. Please see our Supporting Information guidelines for more information: <http://journals.plos.org/plosone/s/supporting-information>.

**Response: We have amended the manuscript accordingly**

Additional Editor Comments:
The reviewers feel that the literature search of this review needs to be updated since the search performed in the current version of the manuscript was done almost one year ago. The reviewers also note that the methodology and discussion need revisions. 

**Response: Thank you for your advice regarding expanding the scope of the literature review. We re ran the search on the 2^nd^ Feb 2023. We have updated the results and discussion accordingly. There were three additional quality papers added. These provided valuable data on the use of portable height/length measurement devices infield, especially in low- and middle-income countries. One of these studies also added depth though including analysis of user perceptions/experiences when using these devices.**

**One additional systematic review was also found through the search. This was *van Gils, R. H. J., Wauben, L. S. G. L., & Helder, O. K. (2022). Body size measuring techniques enabling stress-free growth monitoring of extreme preterm infants inside incubators: A systematic review. PloS one, 17(4), e0267285. https://doi.org/10.1371/journal.pone.0267285*. While this was not directly included in the analysis, the review provided a valuable reference point for comparison and analysis in our discussion. The comparatively significant number of papers published on this topic in 2022 not only enhances our scoping review but also suggests that now is a pertinent time to publish such as review as research into this topic is accelerating.**

**Please see direct responses to reviewers’ comments about methodology and discussion below.**

Reviewers' comments:

Reviewer's Responses to Questions

Comments to the Author

1. Is the manuscript technically sound, and do the data support the conclusions?

The manuscript must describe a technically sound piece of scientific research with data that supports the conclusions. Experiments must have been conducted rigorously, with appropriate controls, replication, and sample sizes. The conclusions must be drawn appropriately based on the data presented.

Reviewer #1: Yes

Reviewer #2: No

Reviewer #3: Partly

**Response: Noted, thanks. Please see direct response to comments/revisions.**

2. Has the statistical analysis been performed appropriately and rigorously?

Reviewer #1: Yes

Reviewer #2: N/A

Reviewer #3: Yes

**Response: Noted, thanks.**

3. Have the authors made all data underlying the findings in their manuscript fully available?

The [PLOS Data policy](http://www.plosone.org/static/policies.action#sharing) requires authors to make all data underlying the findings described in their manuscript fully available without restriction, with rare exception (please refer to the Data Availability Statement in the manuscript PDF file). The data should be provided as part of the manuscript or its supporting information, or deposited to a public repository. For example, in addition to summary statistics, the data points behind means, medians and variance measures should be available. If there are restrictions on publicly sharing data—e.g. participant privacy or use of data from a third party—those must be specified.

Reviewer #1: Yes

Reviewer #2: Yes

Reviewer #3: Yes

**Response: Noted, thanks.**

4. Is the manuscript presented in an intelligible fashion and written in standard English?

PLOS ONE does not copyedit accepted manuscripts, so the language in submitted articles must be clear, correct, and unambiguous. Any typographical or grammatical errors should be corrected at revision, so please note any specific errors here.

Reviewer #1: Yes

Reviewer #2: Yes

Reviewer #3: Yes

**Response: Noted, thanks.**

5. Review Comments to the Author

Please use the space provided to explain your answers to the questions above. You may also include additional comments for the author, including concerns about dual publication, research ethics, or publication ethics. (Please upload your review as an attachment if it exceeds 20,000 characters)

Reviewer #1: This is a timely, interesting, and well written paper. Please check text for minor typos (e.g., line 109, the should be they).

Going through the list of devices outlined in Table 2, most seem to be repurposed digital/laser measuring devices. Some were discontinued and no longer available. A quick search indicates that a person or object's height can be measured with an iPhone. I am not an expert in this specific technology, but I do work in digital app development and my sense is that the field today is far ahead of the technology outlined in the table. Free or low-cost smartphone apps are now available for quantifying numerous body dimensions, although perhaps such software has not been designed for use in young children; but that would be trivial advance.

While I think the authors have done a good job reviewing the available literature, am I right that the digital world has or can move far beyond repurposed digital tape measures for evaluating children's length or height?

**Response: Thank you for taking the time to review this manuscript. It is really valuable to have your perspective as an app developer. As a team of multidisciplinary health care professionals and researchers the perspective you are able to provide is extremely valuable to the translatability and usefulness of this review to industry and product development.**

**In response to your comments:**

**We have reviewed for minor typos and corrected the typo on line 109 accordingly.**

**We also found a number of devices in the grey literatures including apps which could be used to measure body dimensions. However, none of these devices were formally validated to ensure accuracy and precision when used in the paediatric population. While we are not app developers, we agree that this must be a trivial advance to current technology. UNICEF has highlighted the need for technological innovation in height measurement since the target product profile for such devices was released in 2017. We hope this review will highlight this gap, creating an impetus to accelerate advances and promote developers to translate technical improvements seen in other areas to improve measurement and nutritional surveillance of children globally.**

Reviewer #2: This manuscript is an important contribution to the overall quality of the children's anthropometric data collection. However, we would like to suggest some major revisions:

**Thank you for taking the time to review this manuscript. As an anthropometry specialist with decades of experience your insights and opinions are extremely valuable. You have provided very sound feedback that we feel has greatly enhanced and strengthened our review, so thank you for all the thought and consideration you put into writing it. We have addressed individual comments below.**

1. Expend the scope of the literature review up to Dec 2022. Some important articles were recently published and are not included in this review as the authors limited their search to almost 1 year ago (18 January 2022). Few examples:
Bougma et al., American Journal of Clinical Nutrition. 2022 Jul 6;116(1):97-110. doi: 10.1093/ajcn/nqac064.
Jefferds et al., Current Development in Nutrition. 2022 Apr 19;6(6). doi: 10.1093/cdn/nzac085.
Leidman et al., JMIR Biomed Eng 2022;7(2): e40066 doi:10.2196/40066

**Response: Thank you for your advice regarding expanding the scope of the literature review. We re ran the search on the 2^nd^ Feb 2023. We have updated the results and discussion accordingly. In this search we have included the papers suggested above. These provided valuable data on the use of portable height/length measurement devices infield, especially in low- and middle-income countries (LMIC). This in field research is particularly important to ensure that the academic literature is translatable to real world clinical application. This was an area that was not thoroughly discussed prior to inclusion of these papers. Hence expanding the dates of this review added significant depth to the quality of our manuscript. One of these studies also added depth though including analysis of user perceptions/experiences, another important aspect to consider in order to allow for industry transition that was lacking in our prior analysis.**

**One additional systematic review was also found through the search. This was *van Gils, R. H. J., Wauben, L. S. G. L., & Helder, O. K. (2022). Body size measuring techniques enabling stress-free growth monitoring of extreme preterm infants inside incubators: A systematic review. PloS one, 17(4), e0267285. https://doi.org/10.1371/journal.pone.0267285*. While this was not directly included in the analysis, the review provided a valuable reference point for comparison and analysis in our discussion.**

**The comparatively significant number of papers published on this topic in 2022 not only enhances our scoping review but also suggests that now is a pertinent time to publish such a review as research into this topic has accelerated over the last 12 months.**

2. On the performance requirement of the UNICEF TPP, lease have separate section of precision besides the accuracy section included. Include a precision column in Table 2. Also add the term accuracy to the * for mean difference

**Response: Thank you for addressing this. While we thoroughly addressed accuracy, we agree that precision is a very important aspect that was a significant gap in our earlier manuscript.**

**We have included a paragraph on precision following the accuracy section of our results. We have copied it here for your reference:**

***Quantitative data on precision was available for six devices. Only one device, AutoAnthro when tested in the field, reported precision as the mean difference (mm) between measurements using the same device. In this case, the mean difference of 40-77mm did not uphold the ideal UNICEF TPP criteria (23, 24). The other five devices (Leica Disto D2, Bosch GLM40, P2B2D, cannon digital camera and unspecified digital cameras) as well as AutoAnthro when tested in a controlled environment documented precision using technical errors of measurement (TEM) and Bland-Altman plots (6, 16, 19, 20, 26, 28). Each of these studies reported the precision of the device to be comparable to that of manual measurements. However, we could not directly compare these metrics to the UNICEF TPP standards. Kiko, OneGrows and other devices from the grey literature did not provide a formal precision assessments (32, 33).***

**We have also updated Table 2 with a column on precision and added the term accuracy as specified above. We have also included precision as a key focus point in our discussion.**

3. Please nuance the main conclusion about some devices meeting the criteria for accuracy as most were validated in controlled setting. Please refer to the suggested articles in point one. The performance was not consistent in other settings

**Response: Thank you for this advice, we have nuanced the conclusion accordingly. Key changes show that while some devices show promise it is important that devices are designed with purpose and validated within the context that they will be used.**

**We have copied the conclusion here for your reference:**

***This review identified a large gap in the academic literature and product market for portable digital growth measurement devices. Although some devices show promise further research is needed to test the validity of these devices in varying contexts. Continued development and commercialization will be important to improve reliability and validity of these devices for widespread use. Further studies are required to identify and develop devices that meet the UNICEF TPP criteria for height/length measurement devices.***

4. Cost of devices: Please consider the cost of acquisition of the data for the devices not providing directly/immediately the results. See the example of Auto Anthro. Also, considerer the cost recurrent licensing.

**Response: Thank you for this suggestion, this is an important consideration when assessing the cost of such devices. We have updated the results, Table 2 and our discussion accordingly.**

5. Performance requirements: please provide details on each device regarding "Immediate results"

**Response: Thank you for prompting us to clarify this, we have updated Table 2 and our results accordingly. Each device (where data was available) now has a specific time for results listed in seconds. For reference we have defined immediate results as any result taking less than 1 second to process.**

6. References: Please review and reformat all the reference section

**Response: Noted, and references have been reviewed and reformatted according to PLOS ONE guidelines.**

Minor comments:
Introduction
Line 89: include length-for-age also in the definition of stunting, not only height-for-age.

**Response: Amended accordingly.**

Line 91: include weight-for-length in the definition of wasting, not only weight-for-height

**Response: Amended accordingly.**

L91: I am not sure that obesity as defined by weight-for age z-score is a ration.

**Response: We have amended the definition to match that published by the WHO.**

**Definition is copied here for reference. *Obesity: a body mass index greater than 3 standard deviations above the WHO growth standard median.***

L110: Could you specify what you mean by "inconvenient' to use

**Response: Thank you for allowing us to clear up ambiguity around this statement. We have recreated this paragraph to clarify perceived limitations with manual anthropometry. We have copied this paragraph here for your reference:**

***Measurement boards (stadiometers) are currently the gold-standard tool for obtaining child height and length, including in community-based child health surveillance activities (6). While they have been in widespread use for decades, they are cumbersome and heavy.(7) The process of manual data collection also increases the risk of transcription errors, negatively affecting data accuracy.***

Methods:
Line 148: typo. Please proofread all the manuscript

**Response: Amended accordingly. We have also proof read the manuscript for other typos.**

L163: See major comment. Update the scope of the literature review

**Response: Please see response to major comment above. We have amended the dates in the methodology to match this revision.**

L244: typo

**Response: Amended accordingly. We have also proof read the manuscript for other typos.**

Figure 1: please specify "screened by title and abstract

**Response: Amended accordingly.**

Results:
- See major comment for inclusion of separate section on precision.

**Response: Please see response to major comment above.**

- Please be specific on the terms used. Clarify what performance is related to ... accuracy or precision or both and be specific when talking about accuracy versus precision. Do not pull them together under terms like performance.

**Response: Thank you for flagging this important issue. By adding analysis on precision to our results and discussion we were able to highlight the importance of both accuracy and precision to the performance of height/length measurement devices. We also made sure we clearly differentiated between accuracy and precision, ensuring that they were no longer pooled into a single entity.**

Reviewer #3: Overall comments
This study aimed to conduct a scoping review to identify and evaluate portable digital devices that are currently available to assess length and height measurement in children and adolescents <18 years of age. This is an interesting study and informative to understand the current landscape of digital portable devices available for anthropometric measurements. However, there are several key components of the methods are unclear and the interpretation of the findings does not take into account that the evidence from low- and middle-income countries is very little, which warrant discussion and tempering the conclusion in the abstract. I have made several comments below to strengthen the clarity of the methods and interpretation of the results.

**Thank you for taking the time to review this manuscript. You have provided well thought through feedback that we feel has greatly improved and strengthened our review. We found it particularly valuable reflecting on the translatability of these findings to LMICs, where accurate nutritional surveillance of children is paramount. We have addressed individual comments below.**

Major comments
Introduction
o Please clarify how stadiometers or length boards are considered “inconvenient to use”. Many in the field, particularly those working in low- and middle-income country context would consider stadiometers and length boards are the most pragmatic tools to measure height and length. Clarity around how these tools are inconvenient and how digital tools might be 'simple to use' in any given context with varying levels experience with technological devises among users would be important.

**Response: Thank you for allowing us to clear up ambiguity around this statement. We have recreated this paragraph to clarify perceived limitations with manual anthropometry and possible advantages of digitalisation. We have copied this paragraph here for your reference:**

***Measurement boards (stadiometers) are currently the gold-standard tool for obtaining child height and length, including in community-based child health surveillance activities (6). While they have been in widespread use for decades, they are cumbersome and heavy.(7) The process of manual data collection also increases the risk of transcription errors, negatively affecting data accuracy. A digital, accurate measurement device would thus provide significant advantages for clinical assessment and nutritional surveillance of children. If such a device were portable, lightweight, and reliable, with simple data storage and transfer abilities, it would make child health assessments easier. This is especially pertinent in resource-limited settings, where the large, cumbersome measurement board can be particularly difficult to transport and use, and reliable data transcription and record-keeping can be challenging.***

Methods
Study design – please provide a table listing the UNICEF TPP criteria within the study design component of the methods as this is a key component of this study methodology and design. The study design without the specificity of what these 34 criteria are appears incomplete to the reader.

**Response: Thank you for flagging this important issue. We have listed the UNICEF TPP criteria as Figure 1 in the study design section of the methodology. We have also greatly improved this figure by listing in detail a pragmatic definition of how each criterion was defined in our study. These definitions are based directly off the UNCIEF TPP.**

Literature search –
♣ I am surprised that some of the large and interdisciplinary databases, e.g., Pubmed, Web of Science, and Scopus – were not searched as these databases are likely to index most of the public health and clinical literature relevant to this review. Would the authors please clarify the rationale? Including such a rationale in the limitations section, I expect, would be warranted.

**Response: We agree that this is an important consideration which as a team we thoroughly reviewed before commencing our search. We decided on the use of Medline, Embase, CINAHL and Global Health databases following exploratory searches across multiple databases and consultation with an information specialist.** **We also thoroughly cross-checked the references of all papers and systemic or narrative reviews we found in order to ensure that we were not missing any important papers. While we feel that the search was thorough and allowed us to evaluate the most up to date literature on the topic, we do acknowledge the limitations provided by our choice of data bases which we have added to the limitations section of the discussion.**

♣ Please cite Appendix 2 in the text to clarify that the search terms are detailed in the supplementary material.

**Response: Amended accordingly.**

o Lines 186-187: Please provide additional details regarding the Mixed Methods Appraisal Tool for clarity. It is unclear what this tool entails and how it is similar or different from other tools of quality assessment.

**Response**: **We have added a sentence to clarify what the MMAT tool is. We have copied the sentence here for your reference.**

***The MMAT, a tool comprised of two screening questions followed by five questions specific to study type. The MMAT was chosen as it allows for a standardized and objective appraisal of methodological quality across multiple different study types.***

**Further details as to how the MMAT tool is best utilised can be found in the MMAT user guide from MgGill University (reference 14): Hong QN. Mixed Methods Appraisal Tool (MMAT) Version 2018 User Guide2018 3rd April 2022 [cited 2022 5th May 2022]; 1:[11 p.]. Available from:** [**http://mixedmethodsappraisaltoolpublic.pbworks.com/w/file/fetch/127916259/MMAT_2018_criteria-manual_2018-08-01_ENG.pdf**](http://mixedmethodsappraisaltoolpublic.pbworks.com/w/file/fetch/127916259/MMAT_2018_criteria-manual_2018-08-01_ENG.pdf)**.**

o Line 191: Please provide the UNICEF TPP Box 1 earlier, in the study design section for clarify.

**Response: Amended accordingly, please see comment above.**

o Please include either pragmatic definitions of how each criteria in UNICEF TPP Box 1 was operationalized for scoring or use-case examples for scoring each component. It is unclear from the current description, for example, how “long storage life” or “long operational life” or “operates across wide ranging climatic conditions” were systematically scored.

**Response: Amended accordingly, each criterion now has a pragmatic and practical definition based directly off the UNICEF TPP. Please see Figure 1 for details.**

Results:
o Lines 220-221: Please provide additional details regarding the validation designs used in each study. The types of validations and the rigor of validation can vary substantially between studies, therefore such detail will be useful to interpret the utility of these tools. I recommend a table format for clarity (I see that further details are provided in the subsequent two paragraphs generalizing the validation exercises under taken but it is not clear to me which device validation was completed with which design).

**Response: We have created a table (Appendix 2) which directly compares all studies using validation designs. These were compared for rigor, methodolgy and quality using a methodological criterion created by experts in reliability and agreement investigation (reference for this methodology: Kottner J, Audigé L, Brorson S, Donner A, Gajewski BJ, Hróbjartsson A, et al. Guidelines for Reporting Reliability and Agreement Studies (GRRAS) were proposed. Journal of Clinical Epidemiology. 2011;64(1):96-106.)**

o Please also provide additional details regarding the results for the MMAT score – a table in the appendix score each study against the MMAT criteria will be important for clarity.

**Response: We have created a table (Appendix 3) with the results for each component of the MMAT score for each study. This should create clarity around results.**

Discussion:
o Majority of the studies found were developed and validate for use in high-income countries, and only 5 studies, tested in only 3 low- and middle-income countries, that tested the validity of digital portable anthropometry tools. This is a major gap and the very little evidence and development of tools in LMICs suggest that much work is needed here and that the technological barriers post by digital tools, which were not necessarily created with LMIC contexts in mind, certainly do not outweigh the benefits of tools such as the stadiometer. This warrants further comment in the discussion section (i.e. the utility of such tools that have very minimal evidence or validation in LMIC).

**Response: Thank you for this insightful comment. Expanding the dates of the literature search to include an additional 3 papers looking at height/length measurement devices in LMIC contexts reinforces your view. These studies demonstrated that while devices such as AutoAnthro have been validated in controlled environments in high income countries this does not translate when tested in the field in LMICs. This review demonstrates that there is currently a significant design and knowledge gap for translational research within these contexts. This has been addressed in our ammedments to the discussion which we hope creates an impetus for future studies that focus on specific requirements for use in LMICs.**

**We have coppied some ammendments here for your reference:**

***For example, while the AutoAnthro device was accurate and precise in a controlled environment, it did not perform well in a study in clinical contexts in LMICs. Real-world factors such as temperature, lighting, power point connections, clothing specifications for scans and child co-operation need to be accounted for in the design of these products. Until these devices are designed with these operational considerations in mind, they are unlikely to succeed on a larger scale in diverse contexts.***

***…..***

***Given that reliable child growth data is essential to good public health programs, we consider digital devices – if accurate and reliable – to be potentially superior. However, current (or new) products require further development, particularly when considering in context use in LMICs. Currently there are no products that meet the complex requirements for in field use in LMICs, meaning stadiometers remain the gold standard.***

Abstract
o I find the conclusion of the abstract is overstated as the three devises – Leica DistoD2, Authoanthro, and GLM30 – all fail at least one of the UNICEF TPP minimum criteria. As a result, I suggest that no device names be used in the conclusion of the abstract. Rather, I suggest a more balanced and cautious interpretation of the data in the last sentence as follows: “Although some devices show promise, further research is needed to test the validity of these devices in varying contexts, and continued development and commercialization will be important to improve reliability of these devices for widespread use.”

**Response: We have amended the conclusion of the study to be more balanced and cautious as you have outlined above.**

**We have copied the new abstract conclusion here for your reference:**

***While 11 portable, digital devices exist for child height/length measurement, insufficient data are available to fully assess whether they meet the industry’s needs. Although some devices show promise, further research is needed to test the validity of these devices in varying contexts, and continued development and commercialization will be important to improve reliability of these devices for widespread use.***

Minor comments

Introduction
- Please use the definitions of <-2 standard deviations from the reference median when describing stunting and wasting for accuracy and clarity (rather than “low”).

**Response: We have amended this accordingly. All definitions are written as standard deviations from the reference median as per the WHO definition.**

**We have copied the amendments here for your reference:**

***Stunting, defined as more than two standard deviations below the median height-for-age/length-for-age, affects approximately 162 million children under 5 years worldwide.***

***……***

***Wasting is defined as more than two standard deviations below the median weight-for-height/weight-for-length and indicates recent and severe weight loss.***

- Eligibility criteria section: please clarify why the year 1992 was chosen specifically as the start year for searching for relevant articles.

**Response**: **Thank you for this question as we feel that this is an important consideration. As a team this is something we thought deeply about. We searched the literature from 1 January 1992 onwards, which captured 30 years of data. We did this as we believe that 30 years of data would allow for a thorough evaluation of all contemporary product as well as giving us insight into how this has changed over time. We acknowledge that most of the relevant data is from the last decade. However, feel that 30 years of data gave us a good idea of how this field has changed and evolved over time – providing a useful perspective for the review.**
