# Supplementary material for: Portable digital devices for paediatric height and length measurement: A scoping review and target product profile matching analysis
Source: PLoS One. 2023 Jul 26;18(7):e0288995. doi: 10.1371/journal.pone.0288995 (PMC10370750; doi:10.1371/journal.pone.0288995)
Supplement: Attachment — Submitted filename: Response to reviewers_PLOS ONE.docx [file pone.0288995.s004.docx]

**Dear Dr Perumal,**

**Many thanks for your response. We appreciate the time and thought taken to rereview our manuscript. The reviewers have provided valuable feedback which we feel has strengthened our manuscript.**

**Regards,**

**Tasmyn, on behalf of the study team**

Response to reviewers:

Reviewer #1: The authors have made all of my suggested revisions. I have no additional comments. This is a useful review on this topic.

**Response: Thank you.**

Reviewer #4: Thank you for the opportunity to review this scoping review on different portable tools to measure child height and length. The current work describes studies identified through published and grey literature, and how they compare to the UNICEF TPP. Measuring child height and length objectively and with high validity is important, making this an interesting assessment. Enclosed, please find several queries to the authors with the intention of strengthening the manuscript.

**Response: Thank you for your comments. Please find detailed responses below and accompanying revisions in the manuscript.**

Methods:

The search was last conducted >1 year ago (Jan 2022). The researchers should consider re-running the search strategy.

**Response:**

**As per the methods (Page 7), the search was re-run on the 2 February 2023 to ensure the most up-to-date papers were included.**

The search strategy is noted in Appendix, but this does not appear in Methods text. Reference to the corresponding Appendix should be added.

**Response: we have now referenced the corresponding appendix in the methods.**

From reviewing the search strategy, was infant or child length searched? It seems the preference was for height, although the term used for those <2 years is typically length, and child length is noted in the Title of the manuscript.

**Response:** **Thank you for raising this, height and length terminology was comprehensively considered in the development of our search strategy. We are aware that height is measured standing in children >2 years and length is measured lying down for infants <2 years old and we carefully selected available terminology to reflect this in our search. This is demonstrated in our results whereby most studies measured length of children < 2 years, 5 of which exclusively measured length – suggesting this was adequately addressed in the search strategy. Please find these results described as follows:**

***“Three studies (16%) included neonates only, two studies (11%) measured length of children <2 years, and three studies (16%) measured height of children >2 years. Eleven studies (58%) measured both height and length of children”***

In the grey literature searches (Google), why were the first 50 hits considered only? Is there a reference or methodology that supports this approach? Given that Google has strong algorithms that control what a searcher might observe, do the researchers believe this figure is appropriately representative of the tools available?

**Response: Thank you for raising this, we acknowledge that searching google can be overwhelming due to the abundance of information. We are also aware that google search engines use relevancy rankings to bring the most pertinent results to the top.**

**Our search was run in incognito mode with location and trending searches turned off. Our methodology was initially tested using a number pilot run whereby multiple team members searched the grey literature until hits no longer became relevant to the research question. During this pilot it was felt that the first 50 hits captured most of the relevant websites. Furthermore we ran multiple different grey literature searches using different combinations of synonyms which allowed us to adequately capture a breath of resources. We also used snowball searching if any records were identified to be relevant.**

**We have expanded on this in the methodology as follows: *“We also used Google searches to identify any height/length measurement devices that were commercially available at the time of searching. These were run in incognito mode, with location and trending searches turned off. These searches used various combinations of structured search terms and synonyms similar to the formal literature search, and we inspected the first 50 hits. Targeted searches were also conducted on manufacturer websites looking for additional manufacturing information on devices in the academic literature. Snowball searching was also done if any records that were identified appeared relevant.”***

**We do however acknowledge that this approach comes with limitations and as such have included an additional section in our discussion to highlight this *“multiple different combinations of synonyms in our grey literature search found a diversity of results, however we must also acknowledge that reviewing only the first 50 hits would mean that some devices could have been missed”.***

Would it be possible to indicate which reviewers conducted the reviews and which reviewer was approached for consultation?

**Response: Yes, we have added in the initials of the reviewers as follows:**

***“Two reviewers (TS and S Huang) independently assessed titles and abstracts of all retrieved citations. For potentially eligible studies, full texts were retrieved and assessed by two independent reviewers (TS and S Huang) according to the eligibility criteria. Disagreements during both stages were resolved either through discussion or consultation with a third reviewer (AW).”***

Results:

Since the Abstract and Discussion note that 13 portable devices were identified, this should appear within the Results section. It can be determined by counting Table 2, but only that 16 studies were identified is mentioned in the Results. A sentence stating 13 portable devices would improve the manuscript clarity.

**Response: Twelve portable devices were identified, one of which was trialled both in a controlled environment and in the field. An additional sentence stating all these devices has been added to the results, as follows:**

***“Twelve height/length measurement devices were described across the 20 studies (Table 2). These devices included; SCANTIFY, Leica Disto, AutoAnthro, Gulliver, GLM40, Cannon digital camera, unspecified digital camera, P2B2, Optisizer, Coolpix, GLM30 and PePA”***

To assess the UNICEFF TPP, it seems that several characteristics (e.g., cost) go beyond those mentioned as part of in the customized data extraction form. How were these characteristics captured?

**Response:** **Characteristics** **were captured by directly emailing manufacturers and utilising a grey literature search to look for specifications of devices found in the academic literature. This was described in the methods section as follows:**

***“We also used Google searches to identify any height/length measurement devices that were commercially available at the time of searching.... Targeted searches were also conducted on manufacturer websites looking for additional manufacturing information on devices in the academic literature. …We directly emailed manufacturers where further clarification on technical specification was required”.***

Had the researchers come across the following study in their searches?
Additional study:
Baxter, J.-A., Roth, D., Zlotkin, S., Yin, S., Milgram, P. and Mouzaki, M. (2015), Measuring Child Length and Height: Assessing the Accuracy of a Portable Infrared-based Digital Tool. The FASEB Journal, 29: 31.3. <https://doi.org/10.1096/fasebj.29.1_supplement.31.3>

**Response: Yes, this paper did come up in our initial search findings. It is a conference abstract with no corresponding full paper. At the time we contacted the authors to try and find the complete paper without success. The initial decision was made to exclude it on the basis of very limited information. However on reflection, we have amended the manuscript to include it as an abstract only paper. While it doesn’t add much depth to the paper due to very limited detail, adding it highlights a thorough search strategy, which allowed us to provide a solid and evidenced based review.**

For completeness, the researchers should consider adding a simple table to the Appendix that lists the articles excluded and reasons for exclusion. These studies may be of interest to the readership.

**Response: please see included as appendix 5**

Discussion:

Line 284-85: Per above, it is noted that 13 portable devices were identified. This had not previously been noted in the Results, only the number of studies. Suggest adding number of unique devices within the results, as 16 studies is mentioned, and determining that there are 13 devices requires counting lines in the table.

**Response: please see comment above in results section.**

The Neale et al study is introduced in line 310, but then 2 sentences from line 311-16 (starting with UNICEF have identified...) appear before mention of Neale again. Are these 2 sentences supposed to appear here? They seem out of place. As well, being clear about how the methods/scope of the current review is different from Neale would improve the interpretability.

**Response: Thank you we have outlined how our review differs to Neale et al and have revised for clarity as follows:**

***“Recent reviews looking at portable, digital measurement technology for neonates, infants and children have reported similar results. A systematic review by Van Gils et al looked at portable, digital technology for stress-free growth monitoring in preterm infants (38). Like our review, they found that novel digital technologies show high promise, yet current devices need ongoing refinements and improvements in order to meet gold standard requirements, especially within clinical contexts. Similarly, a narrative review by Neale et al assessed recent innovations in portable height/length measurement devices (39), concluding that manual measurement devices such as measurement mats and portable stadiometers remain the preferred measuring tools until digital equivalents can be shown to be accurate and precise. Our study had a wider search strategy scope allowing for numerous new devices to be found compared to the other reviews. However, similar to these reviews, we identified a large gap in the current digital product market, despite devices showing promise none currently meet the UNICEF TPP criteria for an ideal product.”***

Based on the UNICEF TPP findings and last few paragraphs of the Results, could the researchers provide further critical reflection in the Discussion about the practicality of tools available, if they were to be used in diverse settings? (i.e., LMICs) A good portion of space in the Introduction reflects on stunting and wasting, and which are conditions more likely to affect those in LMICs. Do these devices seem practical for use in LMICs? For example, could reflect on cost relative to existing standard tools (could look at seca or Harpenden for cost comparison) or power requirements (standard length and height boards do not currently require a power source).

**Response: Thank you. We touched on this issue in the discussion but have revised for clarity as follows:**

***“Furthermore, current cost of digital portable devices is significantly higher than a stadiometer. Additionally, operational requirements of these conventional methods such as stadiometers not requiring a power source can be considered beneficial, particularly in LMICs.”***

Could the researchers reflect further on the limitations of their own study methods as conducted?

**Response: Thank you we have now added further content to the limitations section of our discussion to the points outlined by the reviewer below:**

(1) Given this is a review of the literature, the researchers likely didn't interact with any of the tools themselves. In other words, without ever seeing the tools, and just reading text, do the reviewers feel that they were adequately able to assess TPP characteristics? Comparing reports of some characteristics in the UNICEFF TPP could be subjective, especially given comparison is being made between studies.

***“During the TPP matching analysis, the reviewers did not test the discussed devices first hand, relying only on the literature for the analysis, limiting the assessment”***

(2) Measuring length and height is quite different, length is conventionally much more difficult to measure - largely related to the age of the child and their cooperation whilst being measured. There is information on length versus height in the Results, so this could be further reflected upon.

***“Given these devices were not directly tested, it was difficulty to extrapolate from the studies the likely added difficulties in measuring length of young children lying down and how this affected device usage.”***

(3) As the UNICEF TPP was published in 2017 and several identified studies were published before this, it is quite plausible that they would not report on such characteristics. This could be reflected on further.

***“Additionally, it must be noted that many studies were written prior to the TPP publication in 2017, making it plausible that these studies may not have considered some specifications desired by the TPP, impacting the devices overall score.”***

Appendix

The Tables presented in the Appendix do not align with the main manuscript (Table 1 in the Appendix is noted as Table 3 in text).

**Response: Amended accordingly. Thank you.**

Search strategy is provided in the Appendix, but not listed in the main paper. Within the Appendix, given the placement of mention of the search strategy, this should appear first (i.e., before UNICEF TPP)

**Response: Amended accordingly. Thank you.**

A table describing exclusions at full-text stage would strengthen the manuscript, and is standard practice for other review platforms

**Response: Amended – added as appendix 5.**
